# The Reaction of Rice Growth to the Arsenic Contamination under Various Irrigation Methods

**DOI:** 10.3390/plants13091253

**Published:** 2024-04-30

**Authors:** Tímea Szalóki, Árpád Székely, Noémi J. Valkovszki, Ákos Tarnawa, Mihály Jancsó

**Affiliations:** 1 Research Center for Irrigation and Water Management, Institute of Environmental Sciences, Hungarian University of Agriculture and Life Sciences, 5540 Szarvas, Hungary; szaloki.timea.palma@uni-mate.hu (T.S.); valkovszki.noemi.julia@uni-mate.hu (N.J.V.); jancso.mihaly@uni-mate.hu (M.J.); 2 Institute of Agronomy, Hungarian University of Agriculture and Life Sciences, 2100 Gödöllő, Hungary; tarnawa.akos@uni-mate.hu

**Keywords:** arsenic stress, rice growing, irrigation effects, genotypic variation

## Abstract

Several studies have explored how arsenic (As) is absorbed and transported in plants, but less attention has been paid to its impact on rice growth and yield in relation to water management. We aimed to assess how arsenic affects plant development under different irrigation methods. The growth and yield parameters of four rice varieties (‘M 488’, ‘Janka’, ‘Szellő’, and ‘Nembo’) in two greenhouse experiments were analyzed in 2021 and 2022 under different irrigation methods (flooded (F), intermittent (I), and aerobic (A)). Three different As concentrations were set up in the soil: 43 mg kg^−1^, 24 mg kg^−1^, and 4 mg kg^−1^. Our results showed that the high As treatment caused severe damage to the plants including leaf yellowing as well as reduced growth and decreased yield parameters. Alternative water management practices such as I and A irrigation could reduce the negative effects of As. At the high level of As stress (43 mg kg^−1^), the I irrigation had the most favorable effect on the yield of ’Janka’ among the tested cultivars compared to the F irrigation (in F: 1.64 ± 1.13 g; in I: 5.45 ± 3.69 g). However, the use of fully aerobic conditions increased the likelihood of drought stress.

## 1. Introduction

Arsenic (As) is a toxic element that contaminates agro-ecological systems in many regions around the world. Its accumulation in crops may pose health risks to animals and humans [1]. The origin of As contamination can be from various sources including natural (rock-forming minerals, groundwater, and geothermal activity) and anthropogenic sources (metal processing, energy production, medicines, waste materials, agricultural products, etc.). A major modern use for arsenic was as pesticides in agriculture [2,3]. Once pesticides enter the soil, pesticide residues and by-products left in the soil can reach the upper part of the plants through the roots [4].

Since rice (*Oryza sativa* L.) is the main agricultural crop in many areas affected by arsenic, particularly in Asia, the accumulation of arsenic in rice is a matter of serious concern in such areas [5]. Different international standards and regulatory limits exist for the presence of As in food and drinking water. The recommended maximum levels (MLs) for inorganic arsenic (iAs) in different types of rice are as follows: 0.20 mg kg^−1^ for white rice; 0.25 mg kg^−1^ for parboiled rice and husked rice; 0.30 mg kg^−1^ for rice waffles, rice wafers, rice crackers, and rice cakes; and 0.10 mg kg^−1^ for rice used in the production of food for infants and young children [6].

As accumulation and speciation in rice depend on both environment and genotype [7]. The As level and As speciation in the grain are influenced by the redox value, pH, phosphate concentration of the soil, the formation of iron plaque in the rhizosphere, microbial activity, and the rice variety [8].

When As enters plants, it can affect many important biological processes, inhibiting plant growth and reducing crop yields. However, different forms of arsenic are not equally harmful to plants [9]. Arsenate interferes with phosphate metabolism processes, such as ATP formation, which leads to lower grain yield (Y) [10]. Arsenite binds to thiol groups of proteins, thus affecting their structures and inhibiting their functions [9]. Treatment with concentrations of 0, 2, 5, and 10 µM of dimethylarsinic acid (DMA) caused rice to suffered from straighthead symptoms, and it induced a strong reduction in fertile percentage (F%) and raised the DMA concentration in husks [11]. Growth on soils containing high levels of total As causes phytotoxic symptoms such as stunted growth, brown spots, and scorching on leaves, which reduce the leaf area and photosynthesis [12,13]. When grown in soil contaminated with 10 mg kg^−1^ As, the yellowing of rice plant leaves and reductions in plant growth and yield parameters were observed [14]. Kaur et al. also observed that the dry weight of the leaves (BMDW) of rice plants grown in soil containing 2.5, 5, and 10 mg kg^−1^ As was reduced compared to the control plants [15]. Under As stress, the plant height (PH), the number of tillers, and the leaf area were reduced. The fresh and dry weights of the plants were also decreased by As treatment [16]. Rice plants supplemented with low (50 μM) and high doses (100 μM) of As(III) showed significant concentration-dependent reductions in growth [17]. A higher As contamination in the soil resulted in higher As accumulation in plant organs [18]. The concentration of As in rice roots was the highest (96%) compared to straw (3%) and raw rice (1%) [19].

Many researchers found significant varietal variations in As uptake and transport [20,21,22,23]. The study of Geng et al. showed cultivar differences in growth after As exposure [24]. Saini et al. demonstrated that the efficiency of As accumulation as well as the As concentration in different parts of the plants varied among different genotypes [25]. Mondal et al. examined arsenite and arsenate stresses on germination and seedlings of different rice varieties. They found that both As forms resulted in a longer germination time and lower germination percentage and reduced root growth. However, treatment with increasing As concentrations above 10 mg L^−1^ increased the fresh root weight. Coleoptile dry biomass was less influenced by As treatment. Different stress responses were detected among the tested cultivars in germination and growth parameters, as well as in As accumulation in rice seedling roots [26].

However, other studies revealed that more significant contributions to the variation in grain As concentration was induced by environmental factors [7,27]. Based on this, some agrotechnical methods were developed for reducing the As content in rice, including water management strategies and the application of different minerals, e.g., phosphorus (P), silicon (Si), sulfur (S), and iron (Fe). These practices can change the As solubility in soil solutions [28].

In addition to arsenic forms, their concentrations, and variety differences, the redox state of the soil is also a key factor affecting arsenic accumulation in rice. The anaerobic conditions in paddy soils support the reductive mobilization of As; therefore, As uptake by rice roots is increased, leading to excessive As accumulation in rice grain and straw [29,30]. In aerobic conditions, As(V) is the dominant form, which is analogous to phosphate (PO_4_^3−^), and one of the mechanisms to uptake As into rice roots is using high affinity PO_4_^3−^ transporters [13]. The bioavailability of As is dramatically reduced in aerobic environments [31,32].

In our previous study, we examined the As content of shoots of four rice varieties in different irrigation systems (flooded and aerobic). No significant differences were found among the varieties in aerobic conditions [33].

A new and emerging technology for producing rice is intermittent irrigation or alternative wetting and drying (AWD), which can combine the simplicity of traditional flooding and the advances of aerobic cultivation such as lower toxic element accumulation and methane emissions [34,35]. AWD methods effectively reduce the total amount of toxic elements including As in rice grains [36,37]. Comparing the drain–flood (aerobic to anaerobic), flood–drain (anaerobic to aerobic), and flood–flood (anaerobic throughout) watering schemes, the levels of arsenic in different tissues of rice plants were highest under the continuously flooded conditions, which was considered to be due to the high availability of arsenic in pore water [38]. Other irrigation treatments included 1. continuous flooding (CF),which consisted of flooding from the fourth leaf to maturity, and 2. interrupted flooding (IF), which was flooding from the fourth leaf to tillering, soil drying for 15 days before the panicle differentiation phase, and subsequent reflooding until the harvest; the researchers found significant year and varietal effects on the yield and As accumulation in rice grains. Under IF irrigation, a significantly lower total As concentration was detected both in the brown and white rice grains compared to the values measured in CF irrigation, but the yield did not decrease [39].

Rice fields contaminated with As also occur in Hungary. There is a lack of research on the As responses of Hungarian rice genotypes and on strategies to reduce As stress through agrotechnological methods. Thus, in our research, we investigated the reactions of widely grown rice varieties of Hungary under elevated As concentrations in the soil combined with different irrigation methods. The aim of our study was to explore the potential positive effect of alternative irrigation methods on the response of rice to arsenic stress and to identify differences in As tolerance between varieties.

## 2. Results

The multivariate analysis of variance (MANOVA) showed that the examined growing and yield parameters were significantly affected by genotype, treatment, irrigation method, and year (Table 1). Their interactions also had significant effects in most cases.

**Figure 1:**
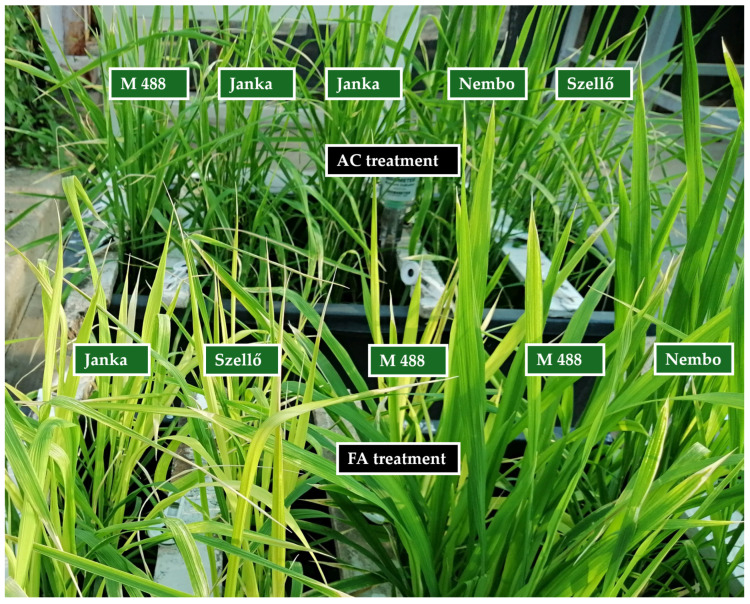
Effects of arsenic on rice leaves two weeks after the As treatment in 2021. FA: flooded irrigation with As treatment; AC: aerobic (rainfed irrigation) control treatment.

The plants’ growth was normal under the control conditions. There were no visible symptoms at an As level of 4 mg kg^−1^. However, the As treatment caused serious damage to rice leaves (Figure 1).

### 2.1. Plant Height (PH)

In general, the irrigation method as well as the arsenic treatment and their interaction had significant effects on PH in both experimental years. Both variety×irrigation and variety×treatment influenced PH, but a variety effect was only detectable in 2022.

**Figure 2:**
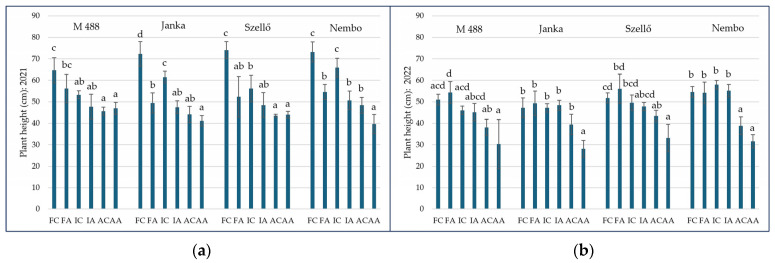
Heights of rice plants in different treatments in 2021 (**a**) and in 2022 (**b**). Based on the amount of water available, the treatments were as follows: FC—flooded control; FA—flooded with As treatment; IC—intermittent flooding control; IA—intermittent flooding with As treatment; AC—aerobic control; AA—aerobic conditions with As treatment. Different letters mean significant differences among the treatments with a *p* ≤ 0.05 according to Tukey (2021) and Games–Howell (2022) multiple range tests.

In the first experiment the average PH values with aerobic (A), intermittent (I), and flooded irrigation were 44.06 ± 3.71, 54.16 ± 7.73, and 62.09 ± 10.97 cm, respectively. In most cases, As treatment reduced the PH except in aerobic irrigation. In the second year, the PHs were 35.43 ± 7.18, 42.65 ± 4.96, and 52.24 ± 5.08 cm, respectively. In contrast to 2021, in 2022, As treatment reduced the PH only with aerobic irrigation. These results are shown in Figure 2. In 2021, the highest PH was measured in the FC treatment for all varieties. ‘M 488’ showed the lowest value with 64.66 ± 5.84 cm; the highest value (74.03 ± 3.93 cm) was measured in ‘Szellő’. Between these two genotypes, the difference was significant; the other two varieties (‘Janka’ and ‘Nembo’) did not show pronounced differences in the FC treatment. The PHs in the IC treatment were significantly lower in ‘Janka’ (61.52 ± 2.70 vs. 72.24 ± 5.72 cm), in ‘Szellő’ (56.19 ± 6.19 vs. 74.03 ± 3.93 cm), and in ‘M 488’ (53.24 ± 1.83 vs. 64.66 ± 5.84 cm) than in FC. In the case of the AC treatment, the lowest values were measured in ‘M 488’ and in ‘Szellő’ (45.59 ± 1.89 and 43.47 ± 0,69), followed by AA; however, in ‘Janka’ and in ‘Nembo’, the PHs were lower in AA than in AC (‘Janka’: 41.18 ± 2.36 vs. 44.15 ± 3.77 cm; ‘Nembo’: 39.65 ± 4.43 vs. 48.37 ± 3.73 cm), and the difference between AC and AA was significant only in ‘Nembo’. Among the control treatments, the differences were significant in ‘Janka’ and in ‘Szellő’ (AC < IC < FC). In ‘Nembo’, AC was significantly lower than IC and FC, although between IC and FC, there was a small difference. In ‘M 488’, the PH was significantly higher in FC than in the other control treatments (Figure 2a).

The As treatments had a negative effect on PH in both IA and FA compared to their control treatments (IC and FC). In ‘Janka’ and ‘Nembo’ the PHs were significantly lower in IA than in IC (‘Janka’: 47.50 ± 3.00 vs. 61.52 ± 2.70 cm, ‘Nembo’: 50.69 ± 4.31 vs. 65.93 ± 4.37 cm). However, the decrease in the other varieties was not remarkable in IA compared to IC (‘M 488’: 47.71 ± 5.78 vs. 53.24 ± 1.83 cm, ‘Szellő’: 48.46 ± 5.80 vs. 56.19 ± 6.19 cm). The FA treatment also caused significant reduction in PH compared to FC in the case of ‘Janka’, ‘Szellő’ and ‘Nembo’. PHs were decreased with 31.52, 29.27 and 25.45%, respectively. In ‘M 488’ the reduction was not significant, only 13.09% (Figure 2a).

In 2022, when the arsenic exposure extended over the entire growing season, the PHs were lower than the previous year, even in control treatments. The aerobic irrigation caused the lowest values in PHs, ranged from8.10 ± 3.89 cm (‘Janka’) to 33.14 ± 6.43 cm (‘Szellő’) in AA and from8.06 ± 3.82 cm (‘M 488’) to 43.39 ± 2.60 cm (‘Szellő’) in AC. In ‘Janka’, significantly lower PH values were measured in AA compared to all other treatments. Similarly, in ‘Nembo’ A treatments caused significant PH reductions. However, in AA the PHs were significantly lower than in FA in all cases. Among IC, IA, FC and FA were not found significant differences in PHs (Figure 2b).

### 2.2. Dry Weight of the Above-Ground Biomass (BMDW)

Similar to PH, lower values of BMDW were measured in the second experimental year than in the first one. The BMDW values were lower with As treatment compared to those in 2021, regardless of the irrigation method and genotype. However, the irrigation method had a stronger impact in 2022 than in 2021. We also found significant variety and treatment × variety interaction effects on BMDW in both years.

Table 2 demonstrates that ‘Szellő’ showed the lowest BMDW values in all treatments with exception of FC in both experimental years. The ‘Szellő’ values were between 17.44 ± 6.88 g (FA) and 33.68 ± 4.76 g (FC) in 2021 and between 9.90 ± 1.14 g (AA) and 20.50 ± 4.11 g (FA) in 2022.

In the case of ‘Janka’, ‘Nembo’, and ‘Szellő’, the BMDWs were the highest in the FC treatment, while ‘M 488’ had the highest value in IC in 2021. Among the treatments, significant differences were observed. In ‘M 488’, the BMDW was significantly lower in the IA treatment than in AA and IC (24.44 ± 7.09 g vs. 42.08 ± 8.46 g and 42.76 ± 12.93 g, respectively), and in ‘Janka’, ‘Szellő’, and ‘Nembo’, there were significant decreases in BMDW values in FA compared to their controls (Table 2).

Within the same treatments, among the varieties, no significant differences were found in most cases, although ‘Szellő’ showed a remarkably lower value than ‘M 488’ in AA (26.74 ± 8.72 g vs. 42.08 ± 8.46 g), and ‘M 488’ had a lower BMDW than ‘Janka’ and ‘Nembo’ in FC (25.06 ± 6.66 g vs. 46.90 ± 12.18 g and 56.00 ± 18.60 g) (Table 1).

In 2022, lower BMDWs were measured under aerobic irrigation (AC and AA), while under the FA and IA treatments, the highest values were detected in ‘M 488’ and ‘Janka’. ‘Szellő’ also had the highest BMDW value in the case of FA, followed by FC, and in ‘Nembo’, the order was IC > FA > FC > IA. However, no significant differences were found between I and F irrigation, regardless of the As treatment and variety. Varietal differences were only detectable between ‘Nembo’ and ‘Szellő’ in the AA treatment, and in the IC treatment, ‘Szellő’ showed a significantly lower value compared to the others (Table 2).

### 2.3. Dry Weight of Roots (RDW)

Under aerobic circumstances, RDWs were usually lower than in anaerobic or periodically anaerobic conditions. In general, the As treatment had no significant effect on this parameter. However, there was a detectable effect in relation to the irrigation method and variety. Variety × irrigation × As treatment (in 2021) and irrigation × As treatment (in 2022) interactions were significant. Among the examined varieties, significant differences were also found in both years with different treatments and irrigation methods.

**Figure 3:**
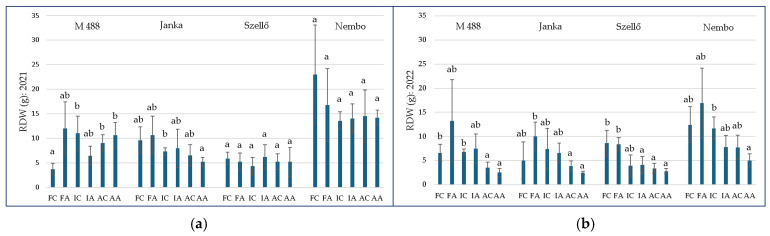
Root dry weight of rice plants under different treatments in 2021 (**a**) and in 2022 (**b**). Treatments: FC—flooded control; FA—flooded with As treatment; IC—intermittent flooding control; IA—intermittent flooding with As treatment; AC—aerobic control; AA—aerobic conditions with As treatment. Different letters mean significant differences among the treatment with *p* ≤ 0.05 according to the Games–Howell multiple range test.

Analyzing the irrigation method along with the As treatment (Figure 3), there were no detectable differences between As-treated plants and their control in all varieties in both years. However, in ‘M 488’, the RDW was significantly lower in FC (3.70 ± 1.19 g) than in the IC (11.04 ± 3.49 g), AC (9.00 ± 1.77 g), and AA (10.62 ± 2.59 g) treatments in 2021 (Figure 3a).

The RDW of ‘Szellő’ in 2021 ranged from 4.36 ± 1.79 g (IC) to 6.20 ± 2.49 g (IA); that of ‘Nembo’ ranged from 12.85 ± 4.36 g (AC) to 22.98 ± 10.08 g (FC). For ‘M 488’, the values were between 3.70 ± 1.19 g (FC) and 12.02 ± 5.38 g (FA) and for ‘Janka’, they were between 5.20 ± 0.92 g (AA) and 10.62 ± 3.89 g (FA) (Figure 3a).

In the same treatments, significant differences were found between the genotypes. In the AA and IC treatments, ‘Szellő’ and ‘Janka’ had significantly lower RDW values than ‘Nembo’. In terms of FC, ‘M 488’ displayed a marked difference from ‘Nembo’ and from ‘Janka’ in 2021.

There were lower RDW values in 2022 than in 2021. The lowest RDWs were measured in AA and ranged from 2.44 ± 0.36 g (‘Janka’) to 5.04 ± 1.36 g (‘Nembo’), followed by the values in the AC treatment. FA produced the highest values, between 8.36 ± 1.38 g (‘Szellő’) and 16.09 ± 7.23 g (‘Nembo’) (Figure 3b). This indicates that the root biomass was slightly increased by As stress under flooded conditions, although genotypic differences may occur. Differences among the treatments within the same variety in 2022 can be seen in Figure 3b.

Similar to 2021, in the next year, the RDW of ‘Nembo’ was the highest in all cases and the lowest values were measured in ‘Szellő’, except in AA and FC, where ‘Janka’ showed slightly lower RDW values than ‘Szellő’. Among the varieties, ‘Nembo’ showed significantly higher RDW values compared to ‘M 488’ and ‘Szellő’ in IC and ‘M 488’ in the AA treatment. In IC, the values in Szellő, ‘M 488’, and Nembo were 3.92 ± 2.24 g, 6.82 ± 0.59 g, and 11.70 ± 2.35 g, respectively; in AA, the RDW of ‘M 488’ and ‘Szellő’ was 2.52 ± 0.88 g and 5.04 ± 1.36 g.

### 2.4. Root Mass Ratio (R%)

The root mass ratio (R%) was calculated as a percentage of root mass over the total biomass (BMDW + RDW).

In general, the R% was lower in aerobic conditions than in other irrigation conditions, although there were significant differences only between the aerobic and flooded irrigation methods in the case of ‘Janka’ and ‘Nembo’ in 2021 and in all examined varieties in 2022. Among the irrigation methods, no remarkable differences were found in ‘M 488’ and ‘Szellő’ in 2021. Between intermittent and flooded irrigation, the difference was significant only in the cases of ‘Nembo’ and ‘Szellő’ in 2022.

**Figure 4:**
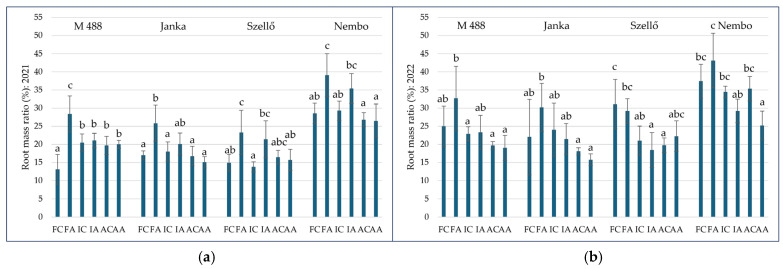
Root mass ratio of rice plants under different treatments in 2021 (**a**) and in 2022 (**b**). Treatments: FC—flooded control; FA—flooded with As treatment; IC—intermittent flooding control; IA—intermittent flooding with As treatment; AC—aerobic control; AA—aerobic conditions with As treatment. Different letters mean significant differences among the treatment with *p* ≤ 0.05 according to the Tukey multiple range test.

As treatment, variety, and irrigation×As treatment interaction had also detectable effects on the R% in 2021. Figure 4 shows the values of the R% in the combined treatments by variety. Under F irrigation, a significantly higher R% value was calculated for the As treatment compared to the control for all genotypes (FA vs. FC). Compared to IC, however, a significant R% increase in the IA treatment was only detectable in ‘Szellő’ (13.80 ± 1.39% vs. 21.39 ± 4.57% in IC and IA, respectively) (Figure 4a). ‘Nembo’ had a significantly higher R% compared to the others in all treatments.

In the following experimental year, there were no significant differences between the control and treated plants within the same irrigation method. However, in FA, a slight increase was detectable in three varieties compared to FC. For example, in ‘Janka’, the R% increased from 22.04 ± 10.40% (FC) to 30.18 ± 6.64% (FA), but because of the high standard deviation, the difference was not statistically significant (Figure 4b). Similar to 2021, in 2022, the highest R% values were observed in ‘Nembo’.

### 2.5. Yield (Y) and Thousand Kernel Weight (TKW)

The yield was influenced by the year, irrigation method, variety, and As treatment. In control treatments, higher average yields were detected in 2021 compared to 2022. In contrast, the yield of As-treated plants was higher in 2022 than in 2021.

Under F and I irrigation, there were higher yields than in A irrigation in the control treatments in both years. However, the As treatment had a negative effect on Y, mainly in 2021. In this year, the yield loss of the As-treated plants was huge within the same irrigation method compared to the control treatment (Table 3). Despite this high reduction, statistically detectable differences were only found in three cases: between FC and FA, a 91.24% yield loss was observed in ‘Szellő’ and a 86.49% loss was measured in ‘Janka’ and between IC and IA, this value was 73.78% in ‘M 488’. Compared to the irrigation methods, the arsenic treatment caused the smallest decrease in Y in A irrigation in three varieties (37.76% in ‘M 488’, 54.17% in ‘Janka’, and 57.34% in ‘Szellő’). The reduction in ‘Nembo’ was the highest between AC and AA (88.22%) compared to FC vs. FA (81.30%) and IC vs. IA (77.74%).

Among the varieties under the F and A treatments, ‘M 488’ had the highest yield; however, the difference was significant in the other varieties only in FC (32.44 ± 10.43 g (‘M 488’) vs. 12.14 ± 4.51 g, 11.87 ± 2.45 g, and 13.08 ± 5.44 g for ‘Janka’, ‘Szellő’, and ‘Nembo’, respectively). In the IC and IA treatments, ‘Janka’ showed the highest yield, followed by ‘M 488’, ‘Nembo’ and ‘Szellő’ (Table 3).

In 2022, no significant differences were found among the treatments; however, with F irrigation, there were higher yields in ‘M 488’. For the other irrigation methods, the yield was in the order of IC < AC < AA < IA. In case of the other genotypes, the I treatment resulted in higher yields than in the F treatment; however, the difference was only significant in ‘Nembo’ (IC vs. F and A treatments) (Table 3). Like in 2021, the lowest yield was measured in the A treatment for all varieties.

The TKW values were also influenced by year, irrigation method, variety, and As treatment. Despite the higher yield, the TKW values were lower in 2021 than in 2022 in most cases (Table 3). The trend of the values was similar in both years within the same variety. The TKW values were the highest in the F treatment, followed by the I treatment in three varieties in both years, although the differences were not remarkable. In ‘Janka’, the highest TKW value was detected in FC, which was significantly higher than in FA (28.74 ± 2.70 g vs. 20.64 ± 3.67 g). In the IC treatment, the TKW was also higher compared to that in IA, but it was not statistically significant (27.07 ± 1.64 g vs. 23.35 ± 2.27 g). Like in 2021, in 2022, the I treatment produced higher values than the F treatment in the case of ‘Janka’. The lowest TKWs were measured in the A treatment in all cases. However, the TKW was significantly lower in the A treatment compared to the F and I treatments only in case of ‘Nembo’ in both years.

### 2.6. Fertile Percentage (F%)

The F% was analyzed as an average of the two experimental years since seasonal variations had no statistically significant effects on this parameter.

**Figure 5:**
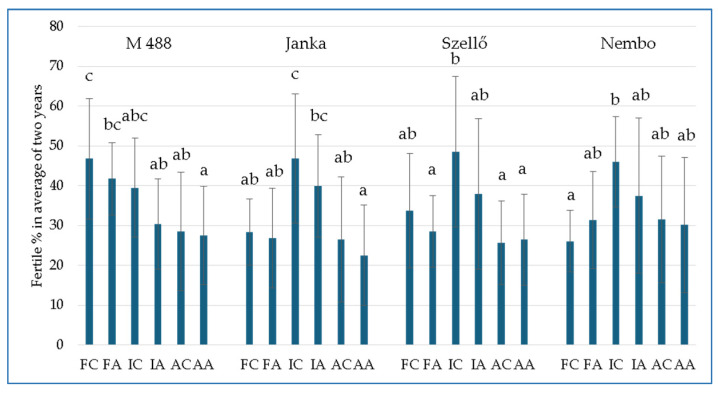
Average rice fertile percentage under different treatments in 2021 and 2022 (n = 10). Treatments: FC—flooded control; FA—flooded with As treatment; IC—intermittent flooding control; IA—intermittent flooding with As treatment; AC—aerobic control; AA—aerobic conditions with As treatment. Different letters mean significant differences among the treatments with *p* ≤ 0.05 according to the Tukey multiple range test.

Figure 5 shows the differences between the treatments within the same variety. The As treatment slightly reduced the F% within the same irrigation methods, but the differences were not significant. In the case of ‘Janka’, ‘Szellő’, and ‘Nembo’, higher F% values were calculated in the I treatment compared to the other two treatments. The F% of ‘Janka’ in IC was significantly higher compared to the values from the F and A treatments (46.82 ± 16.22% vs. 28.35 ± 8.24% and 26.53 ± 15.73%, respectively). For ‘Szellő’, the difference was significant only between the IC and A treatments, while for ‘Nembo’, it was significant between IC and FC (45.97 ± 11.31% vs. 26.07 ± 7 79%).

Significant differences among the varieties were only observed with the F treatment. A significantly higher F% was found in FA between ‘M 488’ (41.72 ± 9.05%) and ‘Szellő’ (28.49 ± 8.98%) and ‘Janka’ (26.84 ± 12.49%). In FC, the F% value of ‘M 488’was also the highest compared to the other varieties (46.77 ± 15.15% vs. 33.79 ± 14.38%, 28.36 ± 8.24%, and 26.07 ± 7.79% for ‘Szellő’, ‘Janka’, and ‘Nembo’, respectively).

### 2.7. Panicle Length (PL)

Similar to the F%, the PL also was analyzed as an average of the two experimental years and year was included as a random factor in the analysis. However, in the case of ‘Janka’, under flooded and intermittent irrigation methods, the As treatment had a significant negative effect on PL compared to their control in 2021 (FC vs. FA: 15.15 ± 0.39 cm vs. 11.65 ± 1.29 cm; IC vs. IA: 14.57 ± 0.94 cm vs. 11.81 ± 0.81 cm). These differences were eliminated in the average of the two years, as shown in Table 3. For ‘Szellő’, the PL was also significantly higher in FC than in FA, even in the average of the two examined years, although between the IC and IA treatments, a remarkable difference was only observed in 2021 (17.47 ± 0.89 cm vs. 13.49 ± 2.95 cm). For the other varieties, there were no large differences in PL between the years and the different treatments.

Varietal differences were observed in all cases. The PL of ‘Szellő’ was significantly higher than that of all the varieties in all treatments. In almost all treatments, the PL of ‘M 488’ was the lowest, followed by ‘Nembo’ and ‘Janka’. In general, the differences between ‘M 488’ and ‘Janka’ were significant except in FA; between ‘M 488’ and ‘Nembo’, the difference in PL was only significant in IC (Table 4).

### 2.8. Principal Component Analyses (PCAs)

**Figure 6:**
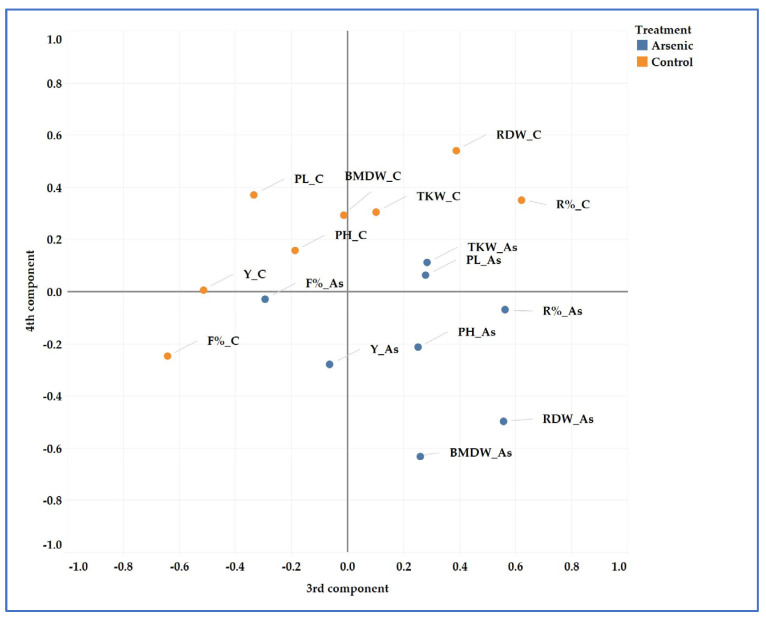
Principal component analysis of the parameters based on two years’ data. PH—plant height; BMDW—above-ground biomass dry weight; RDW—root dry weight; R%—root mass ratio; Y—mean grain yield; TKW—thousand kernel weight; F%—fertile percentage; PL—panicle length.

All the measured and calculated data throughout the two years were analyzed by PCA. The position of the parameters divided by treatments are shown in Figure 6. Five main components were identified in the analyses. These components explained 76.69% of the total variance. The separation between the control and As treatment was significant.

**Figure 7:**
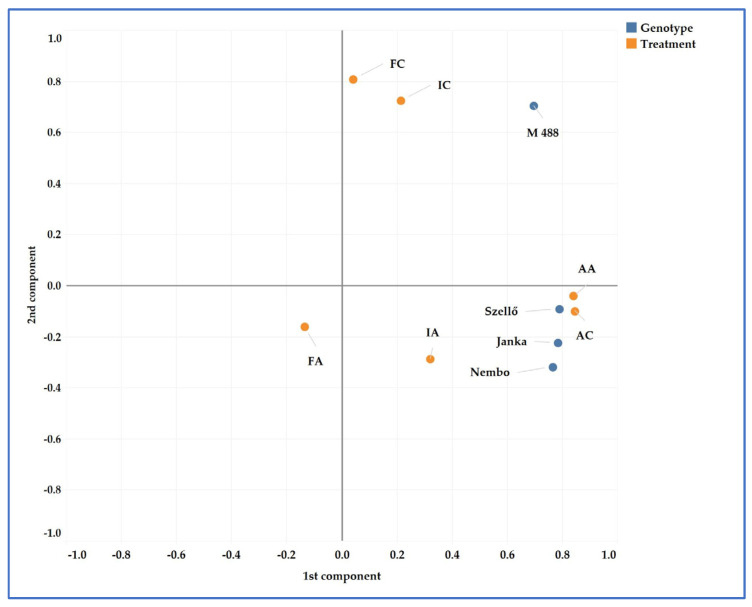
Principal component analysis of the treatments and varieties based on two years’ data. Treatments: FC—flooded control; FA—flooded with As treatment; IC—intermittent flooding control; IA—intermittent flooding with As treatment; AC—aerobic control; AA—aerobic conditions with As treatment.

The analysis of the varieties and the treatments are shown in Figure 7. Two (based on variety) and three components (based on treatment) were identified, and they explained 74.24% (variety) and 64.74% (treatment) of the total variance. The difference between ‘M 488’ and the other varieties was marked. The treatments could be divided into two groups based on the gradient of water availability. The smallest difference between the control and arsenic-treated plants was detected under the aerobic condition (Figure 7).

## 3. Discussion

### 3.1. Effects of As Toxicity on Growth and Yield of Rice

Regarding the effects of As toxicity on rice, reduced growth and yield parameters were detected in our study under flooded and intermittent irrigation mainly in the first experiment, when the As concentration was higher (40 mg kg^−1^), compared to the second experiment (24 mg kg^−1^). Compared to their control, the PH, BMDW, RDW, Y, and TKW values were reduced due to As stress. Similarly, in the research of Abedin et al., the PH, BMDW, RDW, Y, and TKW were significantly reduced with increasing arsenate concentration in the irrigation water. However, in contrast to our results, the RDW reduction was higher or similar to the BMDW reduction [13]. Mondal et al. also found a stronger effect on rice seedling roots than on coleoptile in relation to As stress [26]. The results of Shaibur et al. indicated a higher sensitivity of rice shoots to As than roots in a hydroponic experiment [40]. Similarly, in relation to As toxicity, we observed a higher decrease in the BMDW than the RDW under anaerobic conditions. Therefore, the As treatment increased the R%. Shaibur et al. also found phytotoxic symptoms on rice leaves due to As stress [40], similar to the observation in our experiment, as shown in Figure 1. According to Manawasinghe and Chandrajith, As uptake resulted in yellowish leaves caused by a reduction in chlorophyll content [14].

Azad et al. applied soil containing 0, 1, 5, 10, 20, 30, 40, and 50 mg kg^−1^ As to test the response of rice plants to arsenic in anaerobic conditions. They found significant PH and BMDW reductions with increasing soil As content. Even soil contaminated with 5 mg kg^−1^ and 10 mg kg^−1^ of arsenic caused a significant decrease in the PH and in BMDW, respectively, compared to the control. In a greenhouse experiment, a soil As concentration of 10 mg kg^−1^ caused a significant reduction in the PH, total tiller number, BMDW, PL, 100 KW, and Y compared to the control [14]. However, we only observed a decrease in PH and in BMDW with the As treatment at a concentration of 40 mg kg^−1^, but not with the 24 mg kg^−1^ treatment. Azad et al. also observed a remarkable reduction in yield parameters under As treatment. The grain yield was significantly lower at As concentrations above 20 mg kg^−1^ soil. We only observed a large reduction in yields of rice grown in soil containing 40 mg kg^−1^ As. They found a significant decrease in the TKW with soil concentrations of 40 and 50 mg kg^−1^ As compared to the control treatment, while we measured only a slight decrease in the TKW due to the effect of As. The panicle length also showed a reduction under higher As stress, similar to our findings [41]. In Begum et al.’s study, increasing As contamination resulted in a higher reduction in rice grain yield and straw yield, a reduced TKW, and fewer filled grains per panicle. Compared to the control, the yield loss of rice grown in soil with arsenic concentrations of 15 and 30 mg kg^−1^ was 20.6% and 63.8%, respectively [42]. In contrast, we noticed a remarkable reduction in yield only in 40 mg kg^−1^ As-treated soil. Panaullah et al. observed a BMDW reduction and a 30–33% grain yield reduction in rice grown in 40 mg kg^−1^ As-contaminated soil in a field experiment with a 100 m^2^ plot size [10]. Other studies have also reported a reduction in rice yields due to arsenic toxicity. According to Hossain et al., under flooded irrigation, the RDW and BMDW were significantly affected by arsenic applied to the soil, with a negative correlation between the rate of decline and soil As concentration (4, 19, and 34 mg kg^−1^ As soil concentrations were used in this pot experiment). Similarly, the increasing As content in the soil has also drastically reduced the grain yields. Similar to our experiment, the yield reduction was around 80% with the 34 mg kg^−1^ As treatment [43]. Shri et al. and Abedin and Meharg studied the effect of arsenite (As(III)) and arsenate (As(V)) on rice seedlings and found that the root and the shoot growth of rice seedlings was greatly inhibited by As contamination [44,45]. Vromman et al. evaluated the effect of As on rice seedlings grown in a hydroponic solution and found that the RDW and BMDW were significantly reduced in seedlings grown in a 100 µM As solution compared to the control. Effects on yield-related parameters were also observed. The As treatment strongly reduced the panicle DW and the number of grains per plant. According to the number of filled and sterile grains per plant, despite a significant reduction in the number of fertile and sterile grains, the F% was higher in treated plants than in controls (about 50% vs. 27%) [46]. In contrast, our results showed a decrease in the F% due to the As effects. Tang et al. evaluated the effects of DMA on the rice seed setting rate (F%). They also found that the F% decreased drastically in the treated plants with increasing DMA concentration in the solution [11].

### 3.2. Genotypic Variations

These differences in arsenic sensitivity may be due to the different climatic and soil circumstances, experimental designs, and genotypic variations, as several studies have reported. For example, in the paper of Huq et al., the As response of three boro rice varieties were compared without and with fertilizer application, and in the next season, three aman rice varieties were used. In the first pot experiment, higher RDWs, BMDWs, and grain yields were detected in all cases compared with the second experiment, and the As effect on the plants was also higher due to the continuous irrigation with As-contaminated water. In the next season, aman rice varieties were cultivated in the same pots that were used in the boro season, but no additional As was applied to the pots. Therefore, the As response of rice was moderate. Significant genotypic variations were also detected depending on the treatment [47]. In our study, we also observed differences in growth and yield parameters in the different years. In 2022, there was a hotter and drier growing season, resulting in heat stress and causing lower values for the measured parameters. Among the examined varieties, significant differences were detected. ‘M 488’ had an unexpected response to As stress in relation to the irrigation method, as can be seen in Figure 6. The PH and BMDW did not decrease and the RDW was increased by As in the F treatment in contrast to the other cultivars. However, in the I treatment, arsenic had a stronger negative effect on ‘M 488’ than on the other varieties. Moreover, under F and A irrigation, regardless of the As treatment, ‘M 488’ had the highest grain yield in both growing seasons. Under the I irrigation method, there were no significant differences in mean yield among the varieties. Among the examined varieties, ‘M 488’ appeared to be the most tolerant to the presence of As in the soil in conventional flooded cultivation. In our previous study, the response of ‘M 488’ to salinity stress was also the opposite of that of the other tested varieties, and a negative correlation was found between sodium concentration and yield in all varieties except for ‘M 488’ [48]. Geng compared the response of two winter wheat cultivars to different doses of arsenic and found that the RDW, BMDW, R%, and arsenic concentrations in the plant tissues varied between the two cultivars. For one variety, the BMDW decreased more than the RDW, resulting in a higher R% [24]. We also found that the straw was more sensitive compared to the root; therefore, a higher R% was calculated. Several researchers have reported genotypic variations in As accumulation in rice grain [7,20,21,27]. Pillai et al. showed that yield was negatively correlated with the total arsenic and arsenite content of the rice grain. This indicates that higher As concentrations in grains generally results in lower yields [20].

### 3.3. Water Management Practices and As Uptake

Water management also has effects on As uptake and therefore on As toxicity. Previously, we compered the As accumulation of rice straw under aerobic and anaerobic conditions in a pot experiment. We found that the As concentration of the straw was significantly higher with flooded irrigation than with aerobic irrigation. However, due to the relatively low As concentration in the soil (10.6 mg kg^−1^), no phytotoxic effects on the plants were observed [33]. Several researchers have also reported that aerobic water management practices significantly decreased the total As concentration of rice straw as well as grain compared to anaerobic systems [32,49,50,51]. However, with continuous flooding or intermittent flooding irrigation, a higher yield can be achieved compared with aerobic irrigation. In our study, independent of the variety and As treatment, a higher yield was detected under oversaturated or saturated circumstances than in aerobic conditions. In terms of the PH, BMDW, TKW, and F%, the anaerobic conditions were more favorable than the aerobic ones. In most of the investigated parameters, the As-treated rice plants showed a smaller decline under I irrigation compared to F irrigation. Moreover, the grain yield tended to be similar or even higher under IA than under FA irrigation. Similarly, Spanu et al. also evaluated the effect of three irrigation methods on the As concentration in rice kernels. They found that the As concentration measured in saturation irrigation (the soil was not flooded but it was periodically saturated) was between the values measured in plants grown with flooded and sprinkler irrigation [37]. Carracelas et al. reported that the utilization of intermittent irrigation (AWD) in Uruguay resulted in lower levels of As accumulation in rice grains, which was also significantly affected by the variety [52].

Majumdar et al. also concluded that flood irrigation throughout the rice growing season allows for the maximum productivity and grain yield, which is the primary advantage of anaerobic cultivation. However, in paddy fields with a high As contamination, alternative methods should be used to reduce As uptake in rice and to inhibit the accumulation of arsenic in rice grains [53]. For this reason, intermittent flood irrigation is considered as a good alternative method to reduce arsenic-induced phytotoxicity of rice.

## 4. Materials and Methods

### 4.1. Site Description

The arsenic (As) response of rice was investigated at the Rice Research Station of the Hungarian University of Agriculture and Life Sciences, in Szarvas, in 2021 and 2022.

Szarvas is a small town on the banks of the Körös River, and is the center of Hungarian rice breeding.

### 4.2. Experimental Design and Treatment 

In a greenhouse experiment, twenty-four 90L black pots filled with 50 kg of paddy soil were used. Three Hungarian (‘M 488’, ‘Janka’, and ‘Szellő’) and one Italian (‘Nembo’) *Japonica* rice varieties were used in the experiments. The sowing was carried out on 13 May 2021. Five lines were sowed in every pot. The pot order as well as the rows were randomized. After emergence, 9 plants per row were set up. On the 14th of June, 2.5 g pot^−1^ urea fertilizer was added to each pot, which was equal to 46% active nitrogen. Weed control was performed by hand when it was necessary. In half of the pots, the arsenic treatment was applied with sodium arsenate (Na_2_HAsO_4_ × 7 H_2_O) on 1 July 2021 (49 days after sowing) at the 4–6-leaf stage. The As concentration of the soils were set at 40 mg kg^−1^. This concentration was chosen to represent serious As stress in rice [10,41,43]. In control treatments, the soil of the remained pots contained 4 mg kg^−1^ As (Table 5).

The genotypes and treatments were compared under three irrigation methods (flooded, intermittent flooding, and aerobic conditions) in 5 repetitions. For irrigation, tap water with an undetectable amount of arsenic was used. Aerobic irrigation was based on the field water capacity measured by a basic analog tensiometer (Irrometer Company, Inc., Riverside, CA, USA). In the case of flooded and intermittent flooding irrigation, flooding was carried out the day after the As contamination and the water level was kept 7 cm above the soil surface. For intermittent flooding irrigation, 8 of the 16 flooded pots were drained from July 19 to August 5 and they were kept well-watered for 17 days before reflooding.

The following combinations of treatments were set up:

- Flooded control (FC);
- Flooded with As treatment (FA);
- Intermittent flooding control (IC);
- Intermittent flooding with As treatment (IA);
- Aerobic control (AC);
- Aerobic conditions with As treatment (AA);

### 4.3. The Measured and Calculated Parameters

At the end of the growing season, the plant height (PH), dry weight of above-ground biomass (BMDW), root dry weight (RDW), root percentage (R%), panicle length (PL), the number of fertile (NOFS) and sterile seeds (NOSS) were recorded to determine the fertility % (F%), average yield (Y), and thousand kernel weight (MTKW). TKW was calculated from MTKW at the level of 14% seed moisture (SMC). The BMDW, RDW, and R% were measured row by row; however, the other parameters were measured individually. The calculated row averages were used for further analysis. Plant height was measured with a ruler as the distance between the root neck and panicle neck. For determining the dry weights, the above-ground biomass (stems and leaves) and roots were dried in a Digiheat lab drying cabinet (J.P. Selecta, Barcelona, Spain) until the weights were constants and then were measured using an analytical balance (Sartorius AG, BP 221S, Göttingen, Germany) with accuracy of 0.1 g in five repetitions. R% was calculated with the following equation:

R% = RDW × 100/(BMDW + RDW)

F% was calculated from the NOFS ratio of the total number of seeds (NOFS + NOSS) as follows:

F% = NOFS × 100/(NOFS + NOSS)

MTKW was calculated from the weight (WOFS) and NOFS, and then it was calculated at the level of 14% SMC as TKW:

MTKW = 1000 × WOFS/NOFS TKW = (100 − SMC)/86 × TKW

In 2022, the same pots and soils from 2021 were used. Before sowing, the pots for each treatment were emptied, mixed, and supplemented with 40 kg of paddy soil, and 5 samples from each treatment were taken for analysis. The As concentration of the previously treated soil was 24 mg kg^−1^ and for controls, it remained at 4 mg kg^−1^. Table 5 shows the mean soil parameters of both experimental years. After the pots were refilled, we sowed the rice seeds in a similar procedure as that in 2021 on 23 May 2022. Although the treated plants were exposed to arsenic stress throughout the growing season in this year, the As concentration was lower (23.96 mg kg^−1^) than that in 2021. At the end of the season, the same parameters were calculated.

The basic meteorological data of the two years used were based on the data from an automatic meteorological station (Agromet Solar, Boreas Ltd., Érd, Hungary), which is shown in Table 6.

### 4.4. Data Analyses

Basic mathematical analyses were calculated using Microsoft Excel. Each experiment was carried out under a completely randomized design. The collected data were subjected to two-way analysis of variance (ANOVA) using IBM SPSS statistics software (version 25.0) to confirm the variability and validity of results. The evaluation of the parameters was performed year by year, except for F% and PL. The data were analyzed by the Tukey or Games–Howell multiple range test, according to the Levene’s Test of Equality of Error Variance to determine if there was a significant difference between the treatments and varieties.

After the standardization of the raw data, principal component analyses were performed to check the effect of the parameters, varieties, and treatment. The assumptions of PCA were tested by the Kaiser–Meyer–Olkin (KMO) test and Bartlett’s test. To determine the number of components, an eigenvalue greater than 1 was considered.

## 5. Conclusions

Climatic and soil conditions, cultivation technology, and genotype variation have a great influence on the As response and As uptake of rice. In our study, we focused on the reactions of different rice varieties to As stress under different water management practices. Our results provided information on the strong reduction in growth and yield of rice plants on soils with a high As content. The alternative irrigation methods like intermittent or aerobic irrigation can be effective strategies to alleviate the As toxicity in rice plants. However, it is very important to mention that besides mitigating the negative effects of As, the timing and length of the aerobic period is a crucial aspect in maintaining high rice productivity. In As-contaminated soils, we must grow varieties that are more tolerant to As stress and can be grown without yield loss even with alternative irrigation methods. The tested rice varieties showed different behaviors regarding the phytotoxicity of As and the various irrigation regimes. ‘M 488’ surpassed the other varieties in mean yield in the F and A treatments, regardless of the As level in the soil. The effect of arsenic stress was most effectively alleviated by I irrigation in ‘Janka’, although the yield loss due to the high soil As content was still significant. Considering the water management strategies for reducing the effect of As on rice production, I irrigation may be a good choice along with using adaptive genotypes and timing the water drainage appropriately.

Further studies are needed to find the most accurate timing for intermittent irrigation to reduce As stress without yield loss, and more cultivars should be tested to select As-tolerant genotypes. To avoid other stress factors, controlled climatic conditions should be used to more precisely evaluate the response of plants to As.

## Figures and Tables

**Table 1:** Multivariate analysis of variance (MANOVA) for the significance of the effects of genotype, As treatment, irrigation method, and year on growing and yield parameters of rice.

Variable		SS	df	F	Sig.	Source		SS	df	F	Sig.
Genotype	PH	623	3	10.14	0.00	Genotype	PL	844	3	193.23	0.00
	BMDW	1562	3	10.58	0.00		F%	376	3	0.82	0.48
	RDW	1944	3	56.02	0.00		Y	1137	3	25.55	0.00
	R%	5857	3	104.01	0.00		TKW	1824	3	176.12	0.00
Treat	PH	2448	1	119.47	0.00	Treat	PL	48	1	33.04	0.00
	BMDW	662	1	13.45	0.00		F%	848	1	5.59	0.02
	RDW	8	1	0.65	0.42		Y	999	1	67.37	0.00
	R%	319	1	16.99	0.00		TKW	172	1	49.77	0.00
Irrigation	PH	12,446	2	303.73	0.00	Irrigation	PL	21	2	7.32	0.00
	BMDW	458	2	4.66	0.01		F%	7053	2	23.24	0.00
	RDW	721	2	31.16	0.00		Y	1426	2	48.08	0.00
	R%	1849	2	49.25	0.00		TKW	736	2	106.61	0.00
Year	PH	3426	1	167.21	0.00	Year	PL	10	1	7.12	0.01
	BMDW	11,809	1	239.98	0.00		F%	12	1	0.08	0.78
	RDW	344	1	29.77	0.00		Y	253	1	17.09	0.00
	R%	906	1	48.26	0.00		TKW	165	1	47.73	0.00

SS—sum of squares; PH—plant height; BMDW—above-ground biomass; RDW—root biomass; R%—root mass ratio; PL—panicle length; F%—fertile percentage; Y—yield; TKW—thousand kernel weight.

**Table 2:** The above-ground biomass of rice varieties under different treatments in 2021 and 2022 in a greenhouse experiment.

Variety	Treatment ^1^					
	FC	FA	IC	IA	AC	AA
	BMDW (g): 2021					
‘M 488’	25.06 ± 6.66 ^aA^	28.92 ± 7.85 ^abA^	42.76 ± 12.93 ^bA^	24.44 ± 7.09 ^aA^	36.92 ± 7.07 ^abA^	42.08 ± 8.46 ^bB^
‘Janka’	46.90 ± 12.18 ^bBC^	29.56 ± 4.30 ^aA^	33.80 ± 6.08 ^abA^	30.58 ± 10.50 ^aA^	32.22 ± 7.53 ^abA^	29.34 ± 4.74 ^aAB^
‘Szellő’	33.68 ± 4.76 ^bAB^	17.44 ± 6.88 ^aA^	27.30 ± 10.93 ^abA^	22.88 ± 7.15 ^abA^	26.42 ± 6.72 ^abA^	26.74 ± 8.72 ^abA^
‘Nembo’	56.00 ± 18.60 ^bC^	27.22 ± 14.59 ^aA^	32.88 ± 5.03 ^abA^	25.75 ± 5.63 ^aA^	34.93 ± 10.33 ^abA^	40.36 ± 8.48 ^abAB^
	BMDW (g): 2022					
‘M 488’	19.84 ± 3.17 ^bcA^	24.54 ± 4.94 ^cA^	23.10 ± 2.95 ^cB^	23.66 ± 4.75 ^cA^	14.48 ± 4.20 ^abA^	10.68 ± 3.44 ^aAB^
‘Janka’	16.16 ± 2.73 ^abA^	23.02 ± 5.17 ^bA^	21.62 ± 4.29 ^bB^	23.60 ± 2.64 ^bA^	17.52 ± 4.97 ^abA^	13.08 ± 2.02 ^aAB^
‘Szellő’	18.86 ± 3.81 ^bA^	20.50 ± 4.11 ^bA^	13.88 ± 5.08 ^abA^	17.82 ± 5.16 ^abA^	13.68 ± 3.01 ^abA^	9.90 ± 1.14 ^aA^
‘Nembo’	20.28 ± 2.50 ^abcA^	20.88 ± 4.04 ^bcA^	22.20 ± 4.16 ^cB^	18.64 ± 4.29 ^abcA^	13.86 ± 2.54 ^aA^	14.78 ± 2.06 ^abB^

^1^ Treatments: AA—aerobic conditions with As treatment; AC—aerobic control; FA—flooded with As treatment; FC—flooded control; IA—intermittent flooding with As treatment; IC—intermittent flooding control. Different lowercase letters mean significant differences among the treatments and different capital letters mean significant differences among the varieties with *p* ≤ 0.05 according to the Tukey multiple range test.

**Table 3:** The mean yield (Y) and the thousand kernel weight (TKW) of rice varieties under different treatments in 2021 and 2022 in a greenhouse experiment.

Variety	Treatment ^1^					
	FC	FA	IC	IA	AC	AA
	Mean yield (g) in 2021					
‘M 488’	32.44 ± 10.43 ^cB^	15.34 ± 8.89 ^abcA^	16.54 ± 6.17 ^bcA^	4.34 ± 2.21 ^abA^	3.35 ± 2.44 ^aA^	2.08 ± 1.60 ^aA^
‘Janka’	12.14 ± 4.51 ^bA^	1.64 ± 1.13 ^aA^	17.56 ± 6.41 ^bA^	5.45 ± 3.69 ^abA^	2.40 ± 1.40 ^aA^	1.10 ± 1.47 ^aA^
‘Szellő’	11.87 ± 2.45 ^bA^	1.04 ± 0.66 ^aA^	6.72 ± 3.78 ^abA^	2.35 ± 2.53 ^aA^	1.41 ± 0.80 ^aA^	0.60 ± 0.40 ^aA^
‘Nembo’	13.08 ± 5.44 ^bA^	2.00 ± 1.70 ^aA^	16.40 ± 11.11 ^abA^	3.65 ± 3.26 ^aA^	2.58 ± 2.55 ^aA^	0.30 ± 0.07 ^aA^
	Mean yield (g) in 2022					
‘M 488’	9.02 ± 3.12 ^aB^	11.97 ± 6.27 ^aA^	6.69 ± 2.37 ^aA^	4.70 ± 2.69 ^aAB^	5.55 ± 2.10 ^aA^	4.76 ± 3.83 ^aA^
‘Janka’	2.57 ± 1.47 ^aA^	2.35 ± 2.79 ^aA^	5.09 ± 0.81 ^aA^	5.35 ± 3.25 ^aAB^	4.02 ± 3.38 ^aA^	2.34 ± 2.33 ^aA^
‘Szellő’	3.24 ± 1.80 ^aA^	2.96 ± 1.67 ^aA^	4.87 ± 1.43 ^aA^	3.90 ± 1.02 ^aA^	2.53 ± 1.20 ^aA^	2.73 ± 0.71 ^aA^
‘Nembo’	5.15 ± 2.28 ^aAB^	5.28 ± 2.39 ^aA^	11.98 ± 1.54 ^bB^	10.79 ± 3.65 ^abB^	4.01 ± 2.07 ^aA^	3.72 ± 1.42 ^aA^
	Thousand kernel weight (g) in 2021					
‘M 488’	24.84 ± 0.60 ^cA^	22.22 ± 1.39 ^bcA^	21.93 ± 0.29 ^bA^	19.11 ± 1.50 ^abA^	18.82 ± 2.17 ^abA^	17.74 ± 1.19 ^aA^
‘Janka’	28.74 ± 2.70 ^bAB^	20.64 ± 3.67 ^aA^	27.07 ± 1.64 ^abB^	23.35 ± 2.27 ^abAB^	23.81 ± 2.13 ^abA^	23.98 ± 0.75 ^abC^
‘Szellő’	25.58 ± 0.50 ^bA^	21.32 ± 2.56 ^abA^	20.75 ± 1.56 ^aA^	19.76 ± 4.61 ^abA^	19.34 ± 1.47 ^aA^	20.93 ± 1.00 ^aB^
‘Nembo’	32.39 ± 0.83 ^cB^	29.22 ± 0.18 ^bB^	29.34 ± 0.88 ^bB^	28.39 ± 0.39 ^bB^	21.83 ± 2.27 ^aA^	21.42 ± 0.33 ^aB^
	Thousand kernel weight (g) in 2022					
‘M 488’	24.33 ± 1.28 ^bA^	23.81 ± 1.33 ^bA^	23.53 ± 0.68 ^bA^	21.77 ± 1.24 ^abA^	20.46 ± 0.81 ^aA^	19.39 ± 1.53 ^aA^
‘Janka’	26.81 ± 1.42 ^bA^	24.72 ± 1.75 ^abA^	27.68 ± 1.76 ^bB^	26.54 ± 2.05 ^abB^	26.89 ± 4.19 ^abAB^	21.39 ± 2.05 ^aAB^
‘Szellő’	24.91 ± 0.87 ^bA^	24.08 ± 1.44 ^abA^	23.69 ± 0.84 ^abA^	22.93 ± 0.61 ^abAB^	21.17 ± 1.79 ^aA^	21.17 ± 1.76 ^abAB^
‘Nembo’	33.03 ± 0.92 ^cB^	32.37 ± 1.38 ^cAB^	31.72 ± 0.49 ^bcC^	31.76 ± 1.32 ^cC^	26.16 ± 2.71 ^abB^	25.32 ± 2.09 ^aB^

^1^ Treatments: AA—aerobic conditions with As treatment; AC—aerobic control; FA—flooded with As treatment; FC—flooded control; IA—intermittent flooding with As treatment; IC—intermittent flooding control. Different lowercase letters mean significant differences among the treatments and different capital letters mean significant differences among the varieties with *p* ≤ 0.05 according to the Games–Howell multiple range test.

**Table 4:** Average panicle length of rice varieties under different treatments in 2021 and 2022 in a greenhouse experiment.

Variety	Treatment ^1^					
	FC	FA	IC	IA	AC	AA
	Panicle length (cm)					
‘M 488’	11.99 ± 1.15 ^bA^	10.99 ± 1.19 ^abA^	10.07 ± 0.47 ^aA^	10.02 ± 0.98 ^aA^	10.75 ± 1.19 ^abA^	11.44 ± 0.95 ^bA^
‘Janka’	13.94 ± 1.39 ^abB^	12.35 ± 1.22 ^aA^	13.68 ± 1.20 ^abB^	12.74 ± 1.08 ^abB^	14.64 ± 1.63 ^bB^	13.77 ± 2.01 ^abB^
‘Szellő’	18.74 ± 3.27 ^bC^	15.15 ± 1.78 ^aB^	15.99 ± 1.87 ^abC^	14.56 ± 2.36 ^aC^	16.21 ± 0.97 ^abC^	16.37 ± 2.10 ^bC^
‘Nembo’	13.70 ± 1.12 ^dAB^	12.43 ± 1.15 ^bcdA^	13.24 ± 0.87 ^cdB^	12.14 ± 1.00 ^bcB^	11.43 ± 1.08 ^abA^	10.41 ± 1.04 ^aA^

^1^ Treatments: AA—aerobic conditions with As treatment; AC—aerobic control; FA—flooded with As treatment; FC—flooded control; IA—intermittent flooding with As treatment; IC—intermittent flooding control. Different lowercase letters mean significant differences among the treatments and different capital letters mean significant differences among the varieties with *p* ≤ 0.05 according to the Tukey multiple range test.

**Table 5:** Average soil parameters in 2021 (n = 4) and 2022 (n = 30) before sowing. Arsenic concentration of treated soil after As treatment in 2021 (n = 4) and before sowing in 2022 (n = 15).

Soil Parameter	2021	2022
Soil texture	clay loam	clay loam
Total soluble salts (m/m% dw)	0.16	0.15
Humus (m/m% dw)	1.04	1.71
Carbonated lime (m/m% dw)	0.65	1.73
Nitrate + nitrite N (KCl) (mg/kg dw)	8.30	14.18
Phosphorus (mg/kg dw)	38.63	436.73
Potassium (mg/kg dw)	75.33	123.03
Magnesium (mg/kg dw)	265.50	235.10
Arsenic (HNO_3/_H_2_O_2_) (mg/kg dw) ^1^	3.87	4.10
Arsenic (HNO_3/_H_2_O_2_) (mg/kg dw) ^2^	43.05	23.96

^1^ Average arsenic concentration of control soils. ^2^ Average arsenic concentration of treated soils.

**Table 6:** Open field meteorological data for the two experimental years.

2021	Temperature (°C)			RH ^1^	2022	Temperature (°C)			RH ^1^
	Mean	Max	Min	%		Mean	Max	Min	%
May	14.8	28.8	2.3	76.5	May	18.0	31.4	4.8	67.5
June	22.7	37.5	7.6	65.1	June	23.4	37.6	9.8	60.4
July	25.1	37.5	14.5	67.8	July	24.4	39.9	9.5	56.9
August	21.7	37.2	9.3	70.4	August	24.3	37.6	15.4	66.0
Mean	21.1	35.3	8.4	70.0	Mean	22.5	36.6	9.9	62.7

^1^ RH—relative humidity.

## Data Availability

All data generated or analyzed during this study are included in this published article.
